# Smartphone Apps for Cannabis Cessation: Quality Assessment and Content Analysis

**DOI:** 10.2196/58908

**Published:** 2026-05-14

**Authors:** Siddharth Seth, Sumedha Kushwaha, Reshma Prashad, Michael Chaiton

**Affiliations:** 1 Temerty Faculty of Medicine University of Toronto Toronto, ON Canada; 2 Centre for Addiction and Mental Health Toronto, ON Canada; 3 Institute of Medical Science University of Toronto Toronto, ON Canada; 4 Faculty of Health Sciences Ontario Tech University Oshawa, ON Canada; 5 Dalla Lana School of Public health University of Toronto Toronto, ON Canada

**Keywords:** cannabis, cessation, mobile health interventions, mHealth interventions, mobile apps

## Abstract

**Background:**

Over the past 2 decades, global rates of cannabis use have risen significantly, especially among young adults. This has corresponded to an increase in cannabis-related problems and hospitalizations. Thus, there has been significant interest in developing new interventions that can help facilitate cannabis cessation and reduce hospitalization rates. Specifically, mobile apps have emerged as scalable and accessible stand-alone or adjunct interventions that can help individuals with cannabis use disorders.

**Objective:**

This study aimed to evaluate the quality of free cannabis cessation apps available on both the Apple App Store and Google Play Store, focusing on the analysis of their features, content, and adherence to evidence-based practices.

**Methods:**

A systematic search was conducted in April 2023 using a variety of keywords. The apps were deemed eligible if they were free, available in English, accessible on both the Apple App Store and the Google Play Store, and related to cannabis cessation. Eligible apps were used for at least 1 month and were rated on the Mobile App Rating Scale by 2 reviewers. Interrater reliability was excellent, with a weighted Cohen κ of 0.893 (95% CI 0.835-0.943).

**Results:**

Four apps were included in the analysis, namely, “Grounded–Quit Weed,” “Quit Weed,” “Marijuana Addiction Calendar,” and “Marijuana Anonymous.” The mean overall quality score of the apps was 3.4 out of 5, indicating poor to acceptable quality. The apps scored the highest on the “functionality” section and the lowest on the “information” section. Of the 4 apps, 3 focused on tracking cannabis use and duration of abstinence, whereas 1 focused on peer support. A limited number of cannabis cessation apps were identified, and those that were available were of low quality due to a lack of evidence-based information.

**Conclusions:**

This study is the first to evaluate the current availability and quality of mobile apps designed for cannabis cessation. Unlike previous research that broadly assessed cannabis-related mobile apps, this study focuses on the limited number of free cannabis cessation tools, reflecting what is most available to the general population. The findings highlight a significant gap between the growing demand for virtual cessation tools and the quality of existing options. With the rising global prevalence of cannabis use disorders, there is an increasing need for robust, accessible, and evidence-based therapeutic options. While mobile health apps may be a viable option to support cannabis cessation, the current landscape is limited by poor quality apps and a lack of evidence-based information. From a real-world perspective, this study highlights the need for users to exercise caution when relying on current cannabis cessation apps and underscores the urgent need for the development and evaluation of new evidence-based digital interventions.

## Introduction

On October 17, 2018, Canada legalized recreational cannabis through the Cannabis Act, making it the second country in the world, after Uruguay, to do so at the national level [1]. The act was initially passed to protect public health and safety by restricting youth access to cannabis, preventing inducement to use, reducing illicit production, providing access to a quality-controlled supply of cannabis, and enhancing public awareness of the health risks of cannabis use [2]. However, the legislation appears to have led to a marked increase in cannabis use across Canada [3,4]. In fact, in 2017, the Canadian Tobacco, Alcohol and Drugs Survey reported that the prevalence of past-year cannabis use was 15% [5]. This contrasts sharply with findings from the updated Canadian Substance Use Survey, which reported that 32.4% of the population used cannabis in the past 12 months in 2023 [6]. Furthermore, the 2021 Canadian Cannabis Survey indicated that 17% of Canadians aged 16 years and older reported using cannabis in the past 30 days, with an average consumption of 14.3 days [4]. Another study on cannabis legalization from 2001 to 2019 revealed significant increases in daily cannabis use, past 12-month use, and cannabis-related problems, with the prevalence of cannabis-related problems in Canada rising from 6% to 14% between 2004 and 2019 [7]. In 2019, there were 23.80 million cases of cannabis use disorder globally, with the age-adjusted highest number of incidences observed in high-income North America (121/100,000) [8].

A study combining 19 iterations of the Centre for Addiction and Mental Health Monitor Surveys using a pre-post design found that young adults (aged 18-34 years) had higher rates of cannabis-related problems, such as dependence, anxiety, depressive disorders, and physical health effects, than their older counterparts [7,9]. Although younger individuals continue to exhibit higher levels of cannabis use, the largest rise in consumption since legalization was observed among middle-aged and older adults (those aged 45-64 years and ≥65 years, respectively) [7]. It was concluded that the Cannabis Act was strongly associated with an increased likelihood of cannabis use, daily cannabis use, and cannabis-related problems, including dependence [7]. This finding is supported by Ontario Cannabis Store reports stating that total cannabis sales in Ontario increased by 182% (from 35 million grams to 99 million grams) between the first and second fiscal years [10,11].

According to the Canadian Institute for Health Information, cannabis was the leading contributor to hospitalization rates for substance use among youth aged 10 to 24 years (40%) between 2017 and 2018 [12]. For those aged 25 and older, cannabis was associated with 11% of hospitalizations [12]. Given the age-standardized rate of 104 per 100,000 for youth hospital stays caused by cannabis harm, it is clear that significant resources are required to address youth-specific substance use needs, including timely access to prevention, intervention, and treatment services [12]. Nonetheless, addiction treatment wait times have increased over the last decade, with the average wait time for residential programs reaching 50 days by 2019. Many clients were reported to have been hospitalized, incarcerated, to have attempted suicide, or to have died while awaiting treatment [13].

Various methods can be used to facilitate cannabis addiction treatment, such as cognitive behavioral therapy, which aids individuals in identifying and correcting problematic behaviors, including addiction [14]. Contingency management is another approach that monitors target behaviors and provides or removes tangible rewards to reinforce positive behaviors [14]. Although no medications are currently approved for cannabis use disorder therapy, some are being trialed to help control withdrawal symptoms, and newer clinical trials are focusing on allosteric modulators to inhibit cannabis’s rewarding effect [15].

Mobile phones have revolutionized communication technology, enabling users to access various apps for different purposes. As of 2021, this technology reached a global audience of 6.4 billion individuals and continues to grow, making it pervasive and widely used [16]. Current literature has documented the use of mobile apps for alcohol and tobacco dependence, and research suggests that these apps may promote successful quit attempts among e-cigarette users or vapers [17-19]. The available apps focus on habit tracking, therapy, or education as their primary mode of effectiveness. However, there has been limited research into mobile apps that can be used to facilitate cannabis cessation. This is a pressing issue, as cannabis use is rising globally [20]. In 2014, a content analysis of apps about cannabis on the Apple App Store and the Google Play Store was conducted; however, despite their extensive searching, they only found 1 app that was aimed at promoting cannabis cessation [21]. Since then, the mobile health (mHealth) field has grown significantly, and thousands of new apps have been added to the Apple App Store and the Google Play Store. With the growing field of mHealth, the increasing prevalence of mobile phone use, and the role of technology in disseminating cannabis-related news and information, this study aimed to examine the quality of cannabis cessation apps available in the marketplace, their content and features, popularity among users, and adherence to evidence-based practices. This study primarily focused on free English-language mobile apps available in both the Apple App Store and the Google Play Store.

## Methods

### Search Strategy

Using the search terms “cannabis cessation,” “cannabis quit,” “quit weed,” “marijuana stop,” “stop pot,” “weed cessation,” and “ganja,” the primary and secondary authors searched the Canadian Apple App Store and Google Play Store in April 2023 for smartphone apps targeting cannabis cessation. Searches were conducted directly within the embedded mobile app store platforms rather than their corresponding websites to ensure that the results reflected how users typically discover and download apps.

### Eligibility Criteria

Mobile apps related to cannabis cessation or cannabis behavior modification were included. Apps that were not available for free download or were not in English were excluded. Only free apps were included as they tend to have higher use and accessibility for the general population than paid apps. Apps that were not available on both the Google Play Store and the Apple App Store were also excluded. The Google Play Store and the Apple App Store were selected as the 2 markets of interest, as these platforms are responsible for the largest market share of users [22].

**Figure 1:**
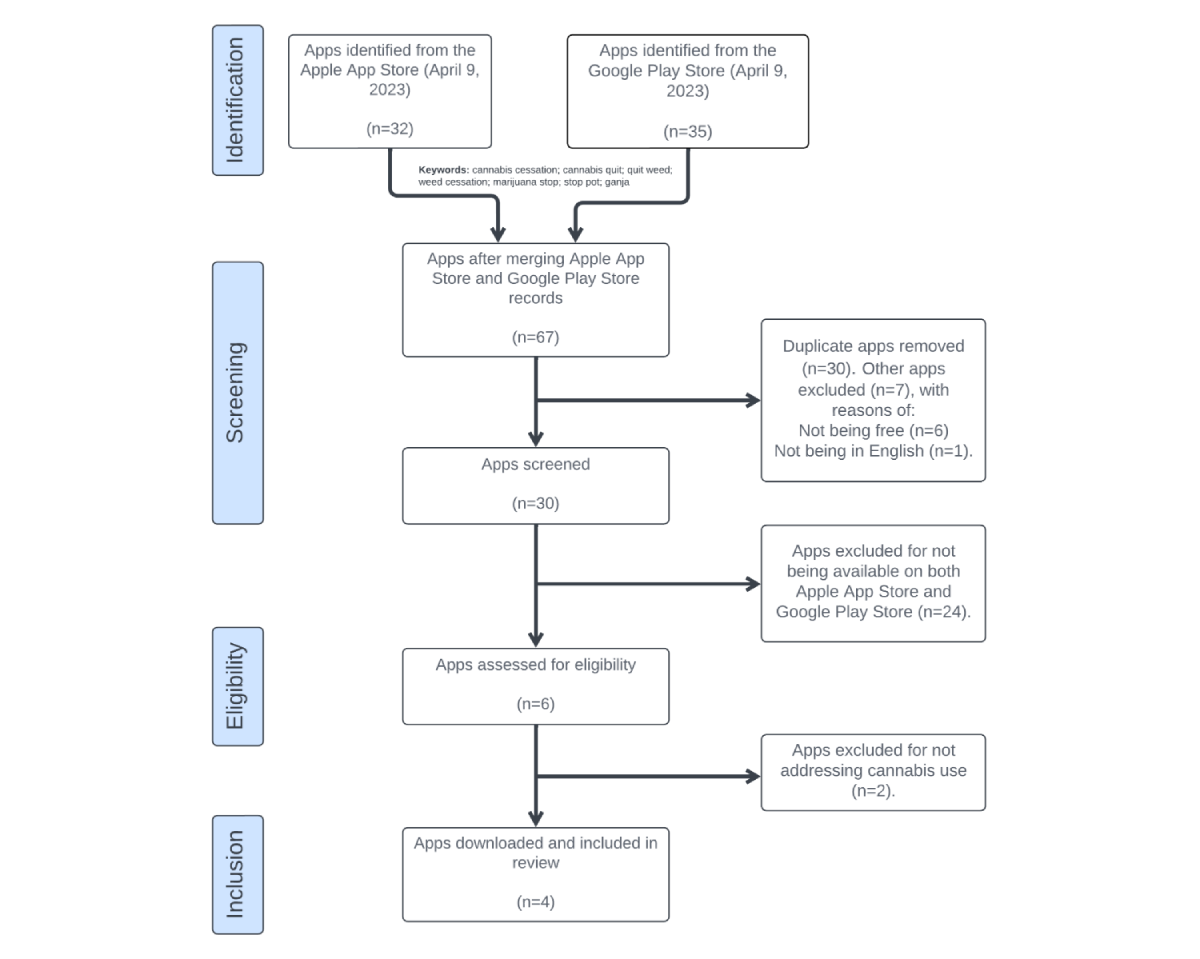
Flow diagram showing the identification, screening, eligibility assessment, and inclusion of cannabis cessation mobile apps for further Mobile App Rating Scale analysis.

Once ineligible apps were removed through a preliminary screening, the remaining apps were examined by checking their descriptions, profiles, relevance to cannabis cessation, and functionality. Those that did not meet the required criteria were excluded. The remaining eligible apps were then independently reviewed by the primary and secondary authors through a 2-pronged screening approach. First, the profiles of the apps were examined on the online app stores, and second, an ancestry search of developer websites, profiles, and overall online presence was conducted when information was available. Apps that were confirmed to have no association with cannabis cessation were excluded, resulting in a final sample of 4 apps. A detailed summary of the inclusion and exclusion process can be seen in Figure 1.

### Assessment of Quality, Contents and Features, and Popularity Among Users

To ensure the accuracy and reliability of our results, each app was rigorously tested over a short period (up to 1 month). This allowed us to classify the apps based on quality, features, and content and to perform the necessary evaluation of these tools. A variety of metrics, including the number of downloads, the number of reviews, and the overall user ratings, were also collected when available. We exclusively assessed the apps on Apple devices. This was because the Apple ecosystem provides a more consistent experience across devices, minimizing the variability associated with different hardware and software configurations found in Android devices.

The quality of each app was evaluated using the Mobile App Rating Scale (MARS), which is a multidimensional measure that is designed to rate the quality of mHealth apps [23]. The MARS has been specifically designed to evaluate mHealth apps and has been validated as a reliable measure of health app quality [23]. The scale comprises 5 categories—engagement, functionality, esthetics, information quality, and subjective quality—with a total of 23 collective items rated using a 5-point scale. For items that could not be adequately assessed, an option of “not applicable” was available. The MARS also includes a subjective quality category; however, it was not factored into the final score.

To identify the strengths and weaknesses of each app, mean scores were calculated for each of the 4 objective categories, while the sum of all 4 category scores provided an overall quality score for each app. However, given the variability among health-related apps, the MARS does not provide a defined threshold for the type of scores a high-quality app should obtain on the scale. The use of the MARS and the overall methodology were based on a previous study that assessed the quality and content of mobile vaping cessation apps [19]. A MARS total mean score of 3.0 was established as the cutoff for apps with acceptable quality [19].

### Data Extraction and Quality Assessment

Two raters tested the MARS with a randomly selected app. Results from the test app showed substantial agreement, with minor differences (≤2 points) that were discussed and resolved. The remaining apps were then independently evaluated and once again showed excellent agreement between the 2 reviewers (≤2 points). The weighted Cohen κ score was 0.893 (95% CI 0.835-0.943).

### Ethical Considerations

This paper does not include any studies involving human participants or animals conducted by the authors; therefore, informed consent was not required. Institutional review board approval, privacy, or compensation statements do not apply to this study. All information is publicly available.

## Results

### Overview

Following a comprehensive screening process, 4 cannabis cessation mobile apps were identified. The full app selection and screening process can be seen in Figure 1.

**Figure 2:**
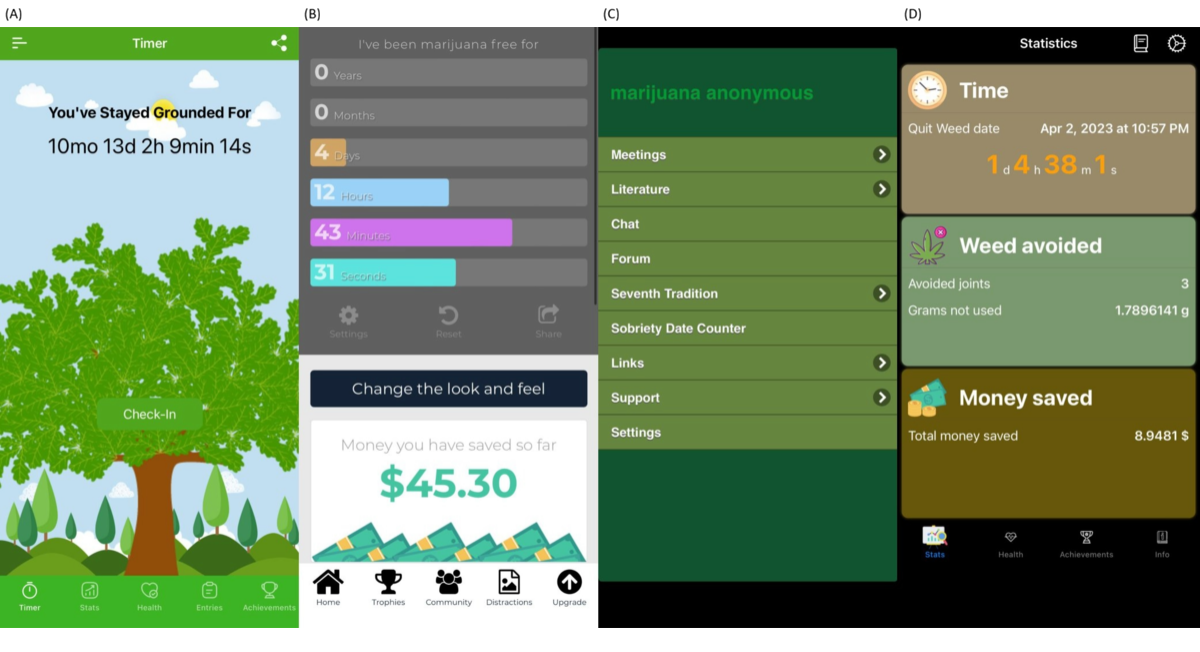
Visual overview of the 4 mobile apps included in the analysis. (A) Grounded–Quit Weed, (B) Marijuana Addiction Calendar, (C) Marijuana Anonymous, and (D) Quit Weed.

On the basis of the inclusion criteria, we selected the following four apps: (1) “Grounded–Quit Weed,” (2) “Quit Weed,” (3) “Marijuana Addiction Calendar,” and (4) “Marijuana Anonymous.” Figure 2 provides a visual overview of the 4 apps included in the analysis.

### General Assessment of Mobile Apps

The overall mean score for the MARS app quality was 3.4, indicating a poor to acceptable quality of the apps. Among the 4 apps, “Grounded–Quit Weed” and “Quit Weed” received the highest score (3.7 each), while “Marijuana Anonymous” received the lowest score (2.8).

On average, the functionality category received the highest mean score (4.3), followed by engagement (3.2), esthetics (3.2), and information (2.8). The full app score breakdown can be seen in Table 1.

**Table 1:** Characteristics of mobile apps based on publicly available information from the Apple App Store and Google Play Store, along with average Mobile App Rating Scale (MARS) scores. The maximum possible average score is 5.

Characteristic	Grounded–Quit Weed	Marijuana Addiction Calendar	Marijuana Anonymous	Quit Weed
App classification	Tracker	Tracker	Support meetings	Tracker
User rating on Apple App Store	4.7	4.4	2.8	4.9
User rating on Google Play Store	4.4	3.1	4.1	4.6
Number of downloads on Google Play Store	>50,000	>10,000	>10,000	>50,000
Engagement	3.6	4.0	1.7	3.4
Functionality	4.3	4.0	4.0	5.0
Esthetics	4.3	2.7	2.3	3.3
Information quality	2.8	2.3	3.0	3.0
MARS total mean score	3.7	3.3	2.8	3.7

### Grounded–Quit Weed

The “Grounded–Quit Weed” was one of the highest scoring apps based on the MARS analysis. It was also the most downloaded app on the Google Play Store. The app began by asking the user a series of questions regarding their cannabis use, specifically about the number of grams used per day, the price per gram, and the quit date. The app also asked users to specify their goal, whether it was quitting cannabis completely, taking a tolerance break, or simply tracking use. The app then used a timer to track the amount of time an individual spent abstaining from cannabis use. Additionally, the app reported the approximate money saved and the amount of cannabis avoided.

The app also featured a journaling component, which allowed users to “check in” by recording whether they used cannabis, the quantity they used, the total amount of money spent, the number and intensity of urges, the type of equipment used, and the date and time of each entry. This enabled users to monitor and analyze the frequency and intensity of their urges to help identify possible triggers. The feature for monitoring withdrawal symptoms and recording detailed trigger information was only available in the paid version of the app and thus was not evaluated. The app included a “Motivation” subsection, which provided users with daily rotating quotes. Additionally, the app included an achievements tracker and a visual representation of user progress, depicted by a tree that grew larger with continued abstinence.

The “Grounded–Quit Weed” app included a “Quit Guide” section that offered limited educational information on cannabis cessation. This section divided the quitting process into 3 distinct phases, providing brief descriptions of each phase, including common side effects, associated thoughts, and guidance on identifying the user’s current stage. However, the app did not provide citations or references to support this phase-based framework. Additionally, the app featured a “Library” section linking to the Grounded420 website, which covered topics such as “What is CBD?” and “What is CHS?” Of these, only the “What is CHS?” paper included citations. Further exploration of the website revealed a range of blog posts on cannabis cessation; however, many of these papers lacked references, which brings into question the credibility and evidence base of the content provided.

The app advertised a community chat feature; however, this was available only in its premium paid version and thus was not evaluated.

### Quit Weed

The “Quit Weed” app used a similar approach to the “Grounded–Quit Weed” app by tracking the time users spent abstaining from cannabis use. Users could enter their average weed use, cost, and quit date in the settings. This app then tracked the approximate amount of cannabis avoided and the approximate money saved. This app also tracked the side effects users may experience during the quitting process and the duration required for those side effects to resolve. While there were references and citations in this section, many were not from peer-reviewed journal papers.

Similar to “Grounded–Quit Weed,” this app had an “Achievements” section, which gave badges based on the number of joints avoided, grams of cannabis avoided, and money saved. The app also seemed to have a motivation section; however, this feature was locked behind a paywall and thus was not evaluated. The app also seemed to have information on recovery phases and associated side effects; however, this content was also blocked behind a paywall and thus was not evaluated.

### Marijuana Addiction Calendar

The “Marijuana Addiction Calendar” also used a timer to track the amount of time users spent abstaining from cannabis use. Similar to “Grounded–Quit Weed”, the app asked for information about the amount of money spent on marijuana per day, the amount of time spent smoking marijuana, the user’s quit date, and the reason for quitting. The app also calculated the money and time saved by not smoking.

The app also had a “Distractions” section, which contained a limited selection of images, sounds, games, and workouts. However, there were only 2 categories of images and 1 category of sounds that were available in the free version. The rest of the images, sounds, and games were restricted to the paid version and thus were not evaluated. The workout section was actually an advertisement for another mobile app and was also not evaluated. Additionally, this app had a “Trophies” section associated with duration of abstinence, ranging from 1 week up to 15 years.

The app also had a community chat feature, which allowed users to post in the forum after signing in with their Apple account. The forum was not very active, with only a few users using this feature weekly. Discussions and posts typically centered on individuals’ initial attempts at quitting; documenting their setbacks; and seeking advice, strategies, and encouragement. Users are also able to like, reply to, or flag other user’s comments as inappropriate. However, users with free memberships were limited in how much they could post. The app also had a secondary feature that allowed users to submit testimonials about the effectiveness of this app on their quitting journey. These testimonials were then displayed to all users. The app also showed frequent full-page advertisements when switching between pages or tabs within the app.

### Marijuana Anonymous

The “Marijuana Anonymous” app functioned primarily as a networking tool to locate licensed Marijuana Anonymous meetings nearby. Although it included a feature to track abstinence duration, this feature was minimal, allowing users to enter only a start date. The app did not provide any educational content on cannabis cessation but instead had a range of documents that described the purpose of “Marijuana Anonymous,” the tenets of the organization, stories from individuals in the organization, and information about their 12-step abstinence process. It was noted that these documents and texts had heavy religious and biblical undertones. Although the primary intended function of the app was to help users locate active meetings, the location-based search feature was nonfunctional at the time of evaluation, limiting its utility. Consequently, the app displayed all scheduled meetings without filtering by user location.

A summary of app features and premium membership pricing can be seen in Table 2.

**Table 2:** App features available in each analyzed mobile app.

Feature	Grounded–Quit Weed	Quit Weed	Marijuana Addiction Calendar	Marijuana Anonymous
Time since cessation	✓	✓	✓	✓
Money saved	✓	✓	✓	—^a^
Time saved by avoiding smoking	—	—	✓	—
Cannabis avoided	✓	✓	—	—
Goal setting	✓	—	✓	—
Withdrawal symptom tracking	✓^b^	✓	—	—
Journaling	✓	—	—	—
Motivation	✓	✓^b^	✓	—
Achievements or trophies	✓	✓	✓	—
Educational content	✓	—	—	✓^c^
Community content (chats or meetings)	✓^b^	✓^b^	✓	✓
Pricing for premium (CAD $1=US $0.75 as of July 2023)	CAD $7.49/month; CAD $35.99/year	CAD $13.99 (one-time payment)	CAD $6.49/month; CAD $52.99/year	—

^a^Not applicable.

^b^Premium content that has not been evaluated.

^c^Educational content is about the Marijuana Anonymous method of quitting rather than about cannabis cessation in general.

## Discussion

### Principal Findings

This is the first study that aimed to evaluate the effectiveness of mobile apps for cannabis cessation and assess the current mobile app market in this regard. The study identified 4 apps available on both the Apple App Store and the Google Play Store for cannabis cessation, namely, “Grounded–Quit Weed,” “Quit Weed,” “Marijuana Anonymous,” and “Marijuana Addiction Calendar*.*” The apps were evaluated using the MARS scale and received an average score of 3.4. The “Grounded–Quit Weed” and the “Marijuana Addiction Calendar” apps received the highest score, whereas “Marijuana Anonymous” received the lowest score. “Grounded–Quit Weed,” “Quit Weed,” and “Marijuana Addiction Calendar” were use tracking apps, whereas “Marijuana Anonymous” was primarily focused on connecting individuals with local Marijuana Anonymous meetings. The results of this study offer valuable insights into the state of the mobile app market for cannabis cessation. Specifically, these results showcase the relative undersaturation of the mobile app field for cannabis cessation. When compared to smoking or vaping cessation mobile apps, it was found that there were significantly fewer apps for cannabis cessation [18,19].

The apps evaluated in this study had significant variability in their comprehensiveness, accessibility, evidence base, and overall quality. While “Grounded–Quit Weed” offered the most extensive range of features, it, like the other apps, lacked appropriate citations and references to support its educational content, limiting the credibility of the information provided. This pattern was reflected in the MARS analysis, in which the apps scored the lowest in the “information” category, suggesting a lack of evidence-based content within the apps. This is likely because clinicians were not involved in the development of these apps, and instead, the apps were focused on creating an engaging and functional experience rather than one that was empirically grounded. This is a critical gap in the field, as recent research has shown that evidence-based mobile apps for behavioral modification, both as stand-alone tools and as part of an intervention, have advantages over standard health interventions [24]. Despite the overall positive correlation between mobile apps and behavioral modifications in the literature, the heterogeneity of results suggests that more research is needed to determine the specific types of mobile apps and features that may yield the most significant benefits for users seeking cannabis cessation support [24].

Use tracking was the most commonly used strategy among both the analyzed and excluded apps. Most apps primarily focused on tracking the duration of cannabis cessation and reporting auxiliary information such as money saved. Although some other features were identified, such as the in-person community meetings associated with the “Marijuana Anonymous” app, a greater variety of strategies and tools is needed within the existing set of apps. Recent research has demonstrated the high efficacy of personalized medical apps, such as artificial intelligence chatbots that adapt to users, in changing health behaviors [25]. The apps should be designed to adapt to users’ moods, preferences, and patterns of use to provide personalized health care support and maximize effectiveness [25]. However, more extensive randomized controlled trials are needed to draw definitive conclusions [25].

### Strengths

This study has several strengths, particularly as the first systematic analysis of cannabis cessation apps. It adds to the growing body of research on alcohol, tobacco, and vaping cessation mobile apps and informs future research and practice in developing and analyzing cessation-focused apps. On the basis of our findings, we propose several recommendations for improving existing apps and developing future apps to better support the cessation process. Specifically, we suggest that researchers focus on providing adequate, evidence-based information for users and offering personalized experiences. Additionally, we recommend the use of multiple tools and methods to help facilitate cannabis cessation, including the use of artificial intelligence and apps that adapt to the users’ moods and preferences [25]. This study lays the groundwork for further research on technology-based interventions, particularly mobile apps and chatbots, to help users quit cannabis.

### Limitations

It is essential to acknowledge the limitations of this study. First, the small sample size limits the generalizability of the findings. However, this also demonstrates the need for further development of mHealth tools for cannabis use disorders. Furthermore, the evaluation of health care mobile apps is a recent endeavor, and thus, additional research is required to develop tools for accurate quality assessments. Additionally, given the rapid pace of mobile app development, it is possible that new apps released after our search were not captured in this review. Only free features were evaluated in this paper, as paid features were beyond the scope of this analysis. However, this approach was justified as free features and apps generally have larger audiences, higher use, and broader accessibility. The search was limited to the Canadian app stores, which could have possibly excluded apps available exclusively in other countries. Finally, the apps were analyzed only on Apple devices and not on Android devices. This meant some apps may not score comparatively on platforms running alternative operating systems. This was especially concerning as the apps were scored lower on the Google Play Store than on the Apple App Store.

### Future Direction

Future research should be done to determine the clinical impact of these apps in regard to different users, ethnicities, and socioeconomic backgrounds. While usability and interface design were evaluated through the “Esthetics” and “Functionality” domains of the MARS analysis, the actual impact of these apps on the end users, particularly in promoting behavior change or supporting cannabis cessation, remains undetermined.

This study is the first to systematically evaluate freely available cannabis cessation mobile apps across major app platforms. Overall, this study found that the effectiveness and quality of currently available mobile apps for cannabis cessation are low, suggesting that more research is needed to develop evidence-based mobile apps for cannabis cessation. Unlike previous studies that focused on any apps associated with cannabis, this study particularly helps identify critical gaps in evidence-based content for mHealth interventions for cannabis cessation [21]. This study directly informs the need for the development of high-quality, evidence-based digital interventions and highlights the need for caution when patients use or clinicians recommend existing cannabis cessation apps. As cannabis use continues to rise globally, the creation and rigorous evaluation of mHealth apps may play a pivotal role in expanding access to effective cessation support and reducing cannabis-related harm.

### Conclusions

This study identified and evaluated 4 mobile apps related to cannabis cessation. On the basis of the MARS analyses conducted, there is a critical gap in the current landscape of cannabis cessation mobile apps. The limited number of available apps, coupled with their low scores in the “information” category, underscores the need for more robust, scientifically tested digital interventions. As cannabis use and cannabis use disorders continue to rise globally, the development of high-quality mHealth tools may prove to be valuable in supporting the cessation process. Therefore, we recommend the development of more robust mobile apps that incorporate strong, evidence-based approaches to cannabis cessation, to better support individuals seeking to reduce or quit cannabis use.

## Abbreviations

MARS: Mobile App Rating Scale
mHealth: mobile health

## Data Availability

Data sharing is not applicable to this study as no datasets were generated or analyzed during this study.
